# Embedding the sustainable development goals in education for the mental health workforce: A scoping review of pedagogies and practice

**DOI:** 10.1111/aphw.70192

**Published:** 2026-07-20

**Authors:** Madeleine Pownall, Ember Corpuz, Richard Harris, Anastasia Ejova, Ziadatul Hikmiah, Ridwan Aji Budi Prasetyo, Anang Sujoko, Ali Mashuri, Dicky C. Pelupessy, Deborah Turnbull, Natasha van Antwerpen

**Affiliations:** ^1^ School of Psychology University of Leeds Leeds UK; ^2^ School of Psychology The University of Adelaide Adelaide South Australia Australia; ^3^ Department of Psychology Universitas Brawijaya Malang Indonesia; ^4^ Department of Psychology Universitas of Indonesia Malang Indonesia

**Keywords:** education, global citizenship, interdisciplinary, SDGs, sustainable development

## Abstract

The United Nations' Sustainable Development Goals (SDGs) provide a global framework for addressing interconnected social, economic, and environmental challenges by 2030. This review examines how the SDGs have been incorporated into interdisciplinary degree and professional training programs for the mental health workforce, broadly defined. The review systematically searched and analyzed the literature, resulting in 47 papers eligible for full‐text assessment. Studies were examined for pedagogical frameworks, teaching strategies, and programmatic interventions explicitly linking SDGs to mental health workforce education. Pedagogical practices were categorized thematically, and the review identified both the educational philosophies underpinning integration efforts and the practical teaching interventions applied. Data were synthesized to describe trends, highlight gaps, and provide insights into approaches for embedding sustainability content in mental health education. Three overarching pedagogical approaches emerged from the reviewed studies: (1) active and critical learning, emphasizing reflective engagement with SDG‐related issues; (2) collaborative learning, fostering interdisciplinary knowledge‐sharing and teamwork; and (3) experiential and service learning, enabling applied practice in community and global contexts. While evidence showed innovative integration of SDGs into curricula, significant gaps remained in consistency and scalability. Challenges included limited programmatic coordination, insufficient training for educators, and a lack of frameworks guiding mental health–specific integration. Overall, findings indicate growing but uneven incorporation of SDGs into education for the global mental health workforce. The review shows how the SDGs can be embedded into mental health workforce education through deliberate and structured pedagogical strategies.

## INTRODUCTION

There is a well‐documented global mental health crisis that both exacerbates and is exacerbated by other global challenges, including climate change, poverty, gender inequality, and threats to sustainability (World Health Organization). To articulate the breadth and urgency of these global challenges, the United Nations member states compiled a list of 17 global goals, the Sustainable Development Goals (SDGs; United Nations, 2015). Each of the SDGs aims to address various social, economic, and environmental challenges facing the world, and has the broad objectives of ending poverty and hunger, promoting quality education and health care, achieving gender equality, ensuring clean water and sanitation, combating climate change, and fostering peace and justice. Each goal has specific targets set to be achieved by 2030, with the overarching aim of creating a more sustainable and equitable future for all people and the planet.

For the SDGs to be achieved, the future generation of the workforce should be equipped with the necessary skills, knowledge, and competencies to contribute to delivering these goals (Pownall et al., 2024). This requires articulating to students and trainees how their professional and disciplinary practice can contribute to achieving the SDGs. Indeed, students are well placed to contribute to the SDGs, as they represent the future workforce (Edwards et al., 2020; Pallant et al., 2020). Further, of 2020, approximately 19.2 million students completed tertiary education (including undergraduate and postgraduate levels) globally (UNESCO, 2020); thus, if these skills, knowledge, insights, and competencies can be harnessed to address the SDGs, this represents a potentially crucial way of addressing the timely global goals.

The integration of the SDGs into educational programs is, therefore, an important endeavor in fostering sustainable development across various sectors (Kioupi & Voulvoulis, 2019). By “integration,” we refer to how the SDGs can form part of vocational, professional, disciplinary, or educational teaching practice. This involves training students about the *relevance* of their discipline to the SDGs and students' capacity to contribute to addressing SDGs and the *importance* of this. In particular, students in interdisciplinary mental health and allied health disciplines (including psychology, health care, medicine) are well‐suited to addressing the SDGs, because of their holistic approach to health, well‐being, and social determinants of health, which forms an integral part of their disciplinary expertise (Badawy & Shaban, 2024; Patel et al., 2018). These disciplines focus explicitly on prevention, early intervention, and the promotion of mental and physical well‐being, which align closely with SDG 3, “Good Health and Well‐Being,” as well as others including SDG 1, “No Poverty,” and SDG 10, “Reduced Inequalities” (Mills, 2018). For example, the British Psychological Society (2024) accreditation standards note explicitly how psychology undergraduate students are well‐placed to contribute to the SDGs, given their disciplinary knowledge and skills. Similarly, SDGs are increasingly integrated in nursing education (Fields et al., 2021) and health care (Badawy & Shaban, 2024). By addressing mental health explicitly, allied health professionals can contribute to reducing health inequalities, fostering inclusive communities, and improving overall quality of life, thereby supporting sustainable development in areas such as education, economic growth, and social justice.

To date, some reviews have considered the integration of the SDGs within higher education curricula (Horey et al., 2018), with particular attention paid to specific disciplines and contexts, including nursing (Badawy & Shaban, 2024), psychology (British Psychological Society, 2024), and engineering (Beagon et al., 2023). Such reviews note the potential for using undergraduate curricula to advance the SDGs; however, there is now scope to examine how pedagogies and practices focused on the SDGs have been integrated into educational programs for students, practitioners, and community leaders known to comprise the “mental health workforce.” Further, there is also scholarship that highlights the need for educators to work across disciplinary boundaries to advance the SDGs (Andreoni & Richard, 2024; Herzig Van Wees et al., 2019; Podgórska & Zdonek, 2024). Yet, this notion has not been comprehensively applied to the context of mental health and allied disciplines.

To date, there is no comprehensive review of how SDGs can be integrated in the mental health workforce. This is important to understand, as there is currently no unified framework, pedagogical set of principles, or standards for how the SDGs may be integrated in interdisciplinary mental health disciplines. The closest concept in the literature is the notion of “global citizenship education” (Pownall et al., 2024), which provides a conceptual framework to consider how students can be mobilized to contribute to the SDGs. Global citizenship education is particularly relevant to mental health training, as mental health professionals routinely work at the intersection of social inequality, human rights, cultural diversity, and global stressors, which are all domains central to global citizenship education. Notably, however, there are varied interpretations of the concept of global citizenship education, and this impacts teaching methods and pedagogies (Horey et al., 2018; Leite, 2022). There have also been other reviews of how global citizenship can be achieved at the subject level (e.g., nursing; Badawy & Shaban, 2024), including specific subject‐level frameworks. For example, in psychology, this has been referred to as ‘psychological literacy’ (see Cranney et al., 2022). Note too that global citizenship education is aligned conceptually with education for sustainable development; global citizenship education typically refers in the literature to the distinct *attributes* and graduate *outcomes* that education for sustainable development seeks to develop.

Therefore, there is now a need to look across connected disciplines of Higher Education to examine inter‐ and intra‐disciplinary pedagogies related to mental health education. The SDGs also require a global perspective and consideration for cultural differences in health‐care provision and Higher Education. Accordingly, we define “mental health” broadly as a state of mental well‐being that enables people to cope with the stressors of life, realize their abilities, learn well, and contribute to their community (WHO, 2022). We, therefore, define “the mental health workforce” in a broad and culturally sensitive way as comprising practitioners in psychology, secondary education, allied health, specialist health, alternative medicine, paramedicine, telehealth, religious pastoral care, and community health (Adebayo et al., 2024). Educational programs for students and practitioners in the mental health workforce are delivered through Higher Education, vocational education, and professional development (WHO, 2022), and all of these contexts will be considered in this review.

Overall, previous research and reviews offer valuable insights into the complex and evolving landscapes of education, mental health, and global citizenship. From addressing practical challenges in integrating global citizenship into university policies (Aktas et al., 2017; Guo, 2014) to examining the multifaceted concept of disciplinary literacies (Beagon et al., 2023), these works provide important perspectives and recommendations for future research and development. However, there is currently a notable lack of synthesis of the literature that looks across disciplines to consider how the SDGs are integrated in all disciplines and subjects that share the goal of advancing mental health provision. Therefore, the aim of this scoping review was to systematically investigate how the SDGs have been integrated into educational programs for all disciplines involved in frontline mental health‐care provision. Our goal was to focus specifically on the teaching methods, frameworks, and pedagogies utilized to address the SDGs, to provide guidance and cohesiveness for future implementation. In this review, integrating the SDGs into education refers to the use of specific pedagogies, teaching methods, and frameworks that enable mental health students to understand how their professional practice can actively contribute to achieving the SDGs, rather than simply learning about them as abstract goals. The research question that guided this review was: *“What is known from the literature about the pedagogies and practice of integrating the SDGs into education for mental health allied disciplines?”*

## METHODS

### Protocol and pre‐registration

Our scoping review protocol was pre‐registered on the Open Science Framework, before screening began (https://osf.io/szdea). We searched the following databases, which all capture content in health research, psychology, humanities, education, and non‐government organization reporting: PubMed, PsycINFO (via Ovid), ProQuest Central, ERIC (via ProQuest), Scopus, The World Health Organization's Institutional Repository for Information Sharing (IRIS), Policy Commons, and PsycEXTRA. The last three databases capture grey literature. We searched for sources that referred to sustainable development goals in the title, abstract, or keywords. An experienced topic librarian checked the logic grids, which can be openly accessed in the Open Science Framework https://osf.io/7xvqe/overview.

Searches combined controlled vocabulary (e.g., MeSH and database‐specific subject headings) and free‐text terms related to (1) pedagogies and educational practices, (2) the SDGs and global literacy concepts, and (3) mental health and allied disciplines. Searches were adapted to the syntax and indexing of each database, limited to English‐language publications, and supplemented by targeted searches of gray literature sources. Example search strings included combinations such as the following: (“education” OR “curriculum” OR “teaching methods” OR pedagogy*) AND (“Sustainable Development Goals” OR SDGs OR “global literacy”) (“mental health” OR psychology OR counselling OR “allied health”) AND (“integrated curriculum” OR “education for sustainable development”) and (“global citizenship” OR “international literacy”) AND (education OR training) AND (mental health). The full search strategy can be openly accessed in the Open Science Framework https://osf.io/7xvqe/overview.

### Eligibility criteria

Table 1 presents our inclusion criteria in full. Our population of interest was the specialized and generalized mental health workforce, broadly defined. We also included “students,” “trainees,” “registrars,” “apprentices,” and “volunteers” in any of these fields. This list was compiled using publicly available sources that provided lists of relevant professional or occupational groups, including the Australian Institute of Health and Welfare (for a list of professions in the mental health workforce), the World Health Organization (for a list of health workforce professions), LibreTexts (for a general list of religious specialists), Wikipedia (for a list of Islamic religious leaders), the US Federal Emergency Management Agency (for lists of Jewish and Hindu religious leaders), and the Government of Manitoba (for a list of Buddhist religious leaders). The full population list is available via the Open Science Framework. The full population can be accessed in the Open Science Framework https://osf.io/7xvqe/overview.

**TABLE 1:** Inclusion and exclusion criteria for the scoping review.

	Include	Exclude
Population	Professionals in health and allied disciplines related to the mental health workforce (e.g., social work) Health students Mental health workers in other areas (e.g., community leaders, religious leaders, first‐responders) Students in first‐response disciplines Volunteers in first‐response domains Teachers and educators *Full list provided in* https://osf.io/7xvqe/?view_only=852af0a2f62d47bd838d9097f2c64bf7.	
Intervention	Actual or proposed training program, course, workshop, seminar, or pedagogical intervention (e.g., classroom activities), as a one‐off activity or program of activities, relating to (a) mental health and (b) one of the sustainable development goals or their targets in a single country or multiple countries (any).	Training/course/workshop not focused on SDGs or their targets. The source can be included if the described intervention focuses on a single country. Training/course/workshop not relating to an aspect of health/well‐being; however, programs (Australian Institute of Health and Welfare, 2023) explicitly and specifically designed for the specialist mental health workers should be included Drug or chemical intervention Descriptive summary of SDG progress and statistics (e.g., a report on country‐level statistics)
Comparator/context	Comparator – any or none Context – as for intervention – one country or multiple, or general	
Outcome	Any or none	
Study characteristics	Primary studies – quantitative or qualitative Reviews – systematic (including umbrella and meta‐analysis), narrative, and qualitative Mixed methods studies Case studies Reports (gray literature or no references) Opinion pieces (gray literature or no references) Study conducted in a single country (any) or multiple countries (i.e., any geographic setting) Theses: Masters, Doctoral, PhD	Studies with non‐human participants Conference proceedings (gray literature) Not written in English Undergraduate or Honours‐level thesis

We were interested in capturing educational interventions, broadly defined. Originally, studies deemed eligible needed to refer to education on *both* SDGs and mental health explicitly, unless they described education for the specialist mental health workforce (e.g., psychologists and psychiatrists), in which case it was sufficient for the program content to relate to SDGs only. However, during screening, many of the papers concerned “health literacy” with a lack of clarity on whether they referred to physical or mental health. Accordingly, we expanded our criteria to capture literature that had a specific focus on the pedagogies used to embed SDGs into training for the future workforce, where it was considered relevant to health and well‐being more broadly defined. All papers included in the final sample had an explicit, identifiable focus on “Sustainable Development Goals.” We included both empirical and non‐empirical studies, so that we could capture literature such as case studies and discussion of pedagogical practice that may not be indexed or referred to explicitly as empirical primary research.

### Search strategy

Our search strategy was designed to identify as many papers as possible relevant to SDG education in our target population. Each harvested paper had to mention SDGs (at least as an acronym), while also describing a pedagogy or practice (e.g., curriculum development), a relevant educational setting (e.g., higher education), and one of the many relevant mental health workforce groups (e.g., primary care). The search was limited to studies published in English in 2015 or later – that is, starting in the year of the ratification of the SDGs. All search terms (which include database‐specific indexing terms) are provided as part of the Supporting Information (https://osf.io/7xvqe/overview).

### Screening procedure

**FIGURE 1:**
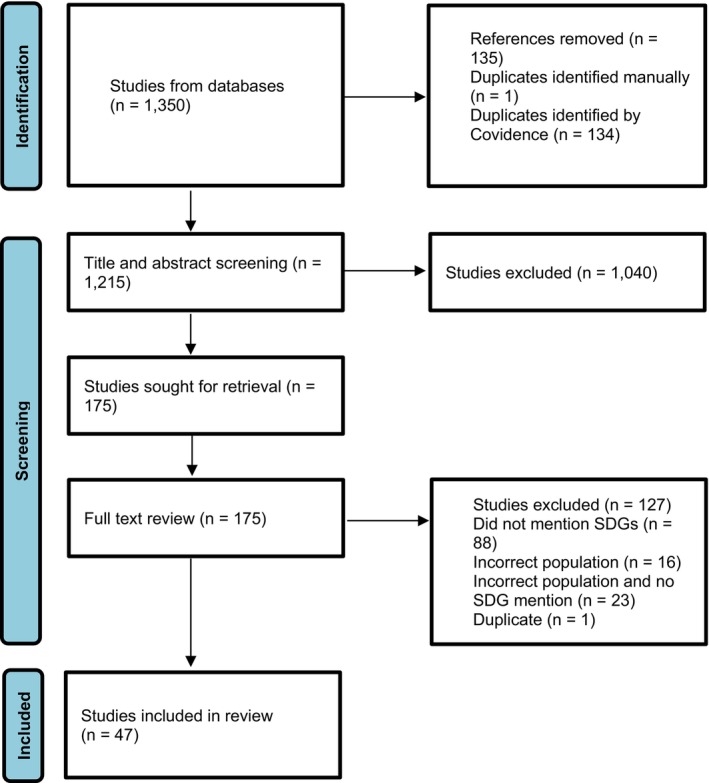
Screening process.

Figure 1 shows our screening process. After concluding the search, we initiated a phased screening procedure. Initially, we screened abstracts and titles (*n* = 1215). Five authors independently evaluated titles and abstracts, with each abstract reviewed by two authors, aiming to identify studies that examined pedagogies on the SDGs either in concert with mental health or in the context of the educational programs for the specialist mental health workforce (see *Protocol and Pre‐registration*). One author reviewed all studies (E.C), providing a common baseline for comparison. Any discrepancies regarding potential inclusions were resolved through adjudication by one of the authors (A.E). Title and abstract screening comprised a single stage. For each source, one person conducted extraction, and 10% of sources were checked for extraction quality by another team member (M.P).

In the subsequent stage of full‐text screening, retrieved papers (*n* = 175) underwent independent evaluation by two authors to determine their suitability for inclusion in the scoping review. Disagreements regarding inclusion were resolved through a consensus process as described previously, with discussion among the team. This led to a sample of 47 final papers. From the final sample, we extracted information and quotes concerning authors and publication date, publication type (including randomized controlled intervention trial, controlled intervention trial, pre–post intervention trial, post‐only intervention trial, systematic/meta‐analytic review, narrative review, report, or opinion piece), methodological approach (qualitative, quantitative, or mixed methods), program sector/industry (tertiary, vocational, professional development, or other with details provided), program location or proposed location (subsequently coded by country, specifying single vs. multiple), program content encompassing aim, coverage of SDGs and their targets, specific modules/topics covered, program format detailing method of delivery (such as face‐to‐face, online, blended, hybrid, training program, course, workshop, seminars), duration in hours over days, and mention of pedagogical strategies and practices (e.g., active learning, adaptive learning, authentic assessment, accelerated formats, e‐portfolios, experiential and case‐based learning, flipped classroom, inclusion and diversity, mind mapping, teamwork and collaboration activities, and universal design for learning approaches), as well as any additional comments on structure/format and proposed/experienced challenges and opportunities regarding SDGs and the global mental health crisis. All categorizations were agreed by at least two authors.

### Analytical approach

We adopted a qualitative content analysis (QCA) approach, informed by Assarroudi et al. (2018), which is an effective method for analyzing large amounts of textual content. The goal of QCA is to uncover and interpret meaning from data, making it particularly suitable for exploring pedagogies and practices for integrating the SDGs. Directed QCA was selected for this study because of its ability to handle large qualitative datasets while providing nuanced, interpretative insights that address gaps in the existing literature (Hsieh & Shannon, 2005). This approach allows researchers to use inductive codes while remaining anchored in prior research (Assarroudi et al., 2018), offering the flexibility needed for this investigation. We approached the analysis inductively and refined our categories as we went. One member of the team (M.P) initially coded the data, and this was checked by another member of the team (A.E) to establish agreement. Any discrepancies in coding were discussed between the reviewers and resolved through consensus, with a final coding agreed jointly.

Then, once all of the data were coded, the rest of the team reviewed the analysis, discussed, and provided feedback and refinements. We used the QCA to interpret the data across aspects of the data extraction table: specifically, we conducted the QCA on the “mention of pedagogical strategies and practices” and “proposed/experienced challenges and opportunities” aspects of the data extraction to address our research question (*What is known from the literature about the pedagogies and practice of integrating the SDGs into education for mental health allied disciplines?)*.

## RESULTS

### Study characteristics

Out of the 47 papers included in the review (see Table 2 for a summary), 20 were literature reviews (predominantly narrative), and 11 were case studies that described and evaluated experiences or learnings from educational initiatives, although only three included limited empirical evaluations. Nine described pre–post intervention trials, one described a controlled trial, and two described the protocol for a pre–post controlled intervention. A further four were reports more generally describing frameworks or motivations for education initiatives, with the final paper being a qualitative study of educators' perspectives on SDGs in education. The full extraction table for all 47 papers can be accessed in the Open Science Framework https://osf.io/7xvqe/overview.

**TABLE 2:** Summary of papers included in review.

ID	Title	Authors	Year	Location	Sector	Population
1	Enhancing mental health pre‐service training with the mhGAP intervention guide: Experiences and lessons learned	Iversen et al.	2021	Not specified	Tertiary	Health‐care providers
2	Project on scaling up midwifery education in four African countries: Final project: Evaluation report	World Health Organization	2021	Maluti, Kanye, Cosenza, Malamulo		Midwives
3	Integrating the social determinants of health into health workforce education and training	World Health Organization	2023	Not specified	Vocational; professional development	Health workforce
4	Why children: Training for health care providers	World Health Organization	2023	Not specified	Professional development	Health‐care providers
5	Optimizing the contributions of the nursing and midwifery workforce to achieve universal health coverage and the sustainable development goals through education, research and practice	World Health Organization	2017	Multiple	Vocational; professional development	Nursing and midwifery professionals
6	Building global nursing citizens through curricular integration of sustainable development goals within an international clinical experience	Morrison‐Beedy et al.	2021	Norway and the United States	Tertiary	Nursing students
7	A web‐based program about sustainable development goals focusing on digital learning, digital health literacy, and nutrition for professional development in Ethiopia and Rwanda: Development of a pedagogical method	Balter et al.	2022	Ethiopia and Rwanda	Professional development	Participants from various ministries
8	An authentic learner‐centered planetary health assignment: A five‐year evaluation of student choices to address sustainable development goal 13 (climate action)	McLean et al.	2022	Australia	Tertiary	Medical students
9	Looking toward 2030: Strengthening midwifery education through regional partnerships	Srisaeng & Upvall	2020	Thailand and Laos	Tertiary; vocational; professional development	Midwives and nurses
10	Child health nurses in Solomon Islands, piloting the “Bachelor of nursing: Child health”	Burhin et al.	2023	Solomon Islands	Tertiary	Nursing students
11	Enlightening and empowering students to take action: Embedding sustainability into nursing curriculum	Fields et al.	2023	Australia	Tertiary	Nurses
12	Global citizens, healthy communities: integrating the sustainable development goals into the nursing curriculum	Upvall et al.	2019	United States	Tertiary; College of Nursing	Nurses
13	Considering developmental concepts from attachment theory to inform graduate student training in global trauma and disaster psychology	Fox et al.	2018	United States	Other: Graduate program/graduate student trainees	Psychology graduate students
14	Taking child development outside of the textbook, the university, and the country: Utilizing the UN's sustainable goal academy in an upper‐level developmental psychology seminar	Zosh	2021	Not specified		Psychologist
15	Teaching sustainable development goals and social development: A case study teaching method	Addo et al.	2022	Not specified	Tertiary; vocational	Social workers
16	SDG's and systems science: teaching beyond the micro–macro divide in social work education	Kaloga et al.	2022	United States	Tertiary	Social work students
17	SDG‐focused social work education in Georgia	Shatberashvili & Sadzaglishvili	2022	Georgia	Tertiary; MA social work program	Social workers
18	Obulala Na‐Maani: Unity is strength: Speech‐language therapy and community engagement in three Kenyan communities	Staley et al.	2023	Kenya	Vocational	Speech‐language therapists
19	Facilitating faculty development for training in multicultural competence in health service psychology graduate programs through an international collaboration	Gopal et al.	2023	United States and India	Tertiary	University faculty members
20	Virtual study‐abroad through web conferencing: Sharing knowledge and building cultural appreciation in nursing education and practice	King et al	2021	United States and Norway		Nursing students
21	A case study on the impact of a web‐based animated storyline module for global health pedagogy: Student perspectives	Ezezika & Jarrah	2022	Canada	Tertiary;	Health studies students
22	Interventions using the Qur'an to promote mental health: A systematic scoping review	Owens et al.	2023	Various		Non‐specific
23	University engagement in achieving sustainable development goals: A synthesis of case studies from the SUEUAA study 1	Neary & Osborne	2018	Glasgow; Harare; Dar‐es‐Salaam; Johannesburg; Duhok; Sanandaj; and Manila		University faculty members
24	The role personal responsibility norms play in sustainable development for university students: The impact of service‐learning projects	Mangas et al.	2021	Spain	Tertiary	Degree in primary education and the degree in social education
25	Adolescent health in the sustainable development goal era: Are we aligned for multisectoral action?	George et al.	2021	Low–middle income countries		Health and youth leaders
26	Health professional workforce education in the Asia Pacific	Lees et al.	2016	Australia	Tertiary	Healthcare professionals
27	Substantiation of the advanced training program “social work with military personnel and military‐social work in the context of sustainable development goals”?	Trubavina et al.	2021	Ukraine	Vocational	Social workers and military social work
28	Evaluation of emergency first response's competency in undergraduate college students: Enhancing sustainable medical education in the community for work occupational safety	Dieck‐Assad et al.	2021	Mexico	Tertiary; vocational;	Emergency First Responders
29	Indigenous knowledge and social work education in Nigeria: Challenges and need for sustainable development	Nnama‐Okechukwu et al.	2023	Nigeria	Tertiary	Social work educators, students, practitioners
30	Volunteer service and service learning: opportunities, partnerships, and united nations millennium development goals	Dalmida et al.	2016	United States	Vocational; professional development	Nurses
31	Service‐learning projects in university degrees based on sustainable development goals: Proposals and results	Castro et al.	2020	Spain	Tertiary	Students
32	Internationalizing nursing curricula in a rapidly globalizing world	Kunaviktikul & Turale	2020	Not specified	Tertiary; vocational; professional development	Nurses
33	Disaster risk reduction education: Tensions and connections with sustainable development goals	Cabello et al.	2021	Various	Vocational; professional development	Approaches to education about DRR with the target group, children
34	Approach Developed According To Sustainable development goals and challenges for future professionals in social intervention	Picado‐Valverde et al.	2022	Spain	Tertiary	Teachers
35	Sustainable development perspectives in physical education teacher education course syllabi: An analysis of learning outcomes	Froberg & Lundvall	2022	Sweden	Other: Teachers in primary and secondary school	Physical education teachers
36	Development, application and evaluation of an active learning methodology for health science students, oriented towards equity and cultural diversity in the treatment and care of geriatric patients	Sanchez De Miguel et al.	2022	Spain	Tertiary	Psychologist; psychology, nursing and dentistry students
37	Education on the sustainable development goals for nursing students: Is Freire the answer?	Fields et al.	2022	Australia	Other: Not specified	Not specified
38	Implementing the sustainable development goals at university level	Albareda‐Tiana et al.	2018	Spain	Tertiary	Academic staff
39	At a crossroads: How can Nepal enhance its community health care system to achieve sustainable development goal 3 and universal health coverage?	Schwarz et al.	2020	Nepal	Vocational; professional development	Community health workers
40	Achievements and challenges for higher education during the COVID‐19 pandemic: A rapid review of media in Africa	Sonn et al.	2021	Africa	Tertiary; vocational	Not specified
41	Expert consensus on core topics of sustainable development online learning module for family physicians: A Delphi study	Suvarnabhumi et al.	2022	Thailand	Tertiary; professional development	Family medicine educators
42	Teacher professional development, character education, and well‐being: Multicomponent intervention based on positive psychology	Garcia‐Alvarez et al.	2023	Uruguay	Tertiary; professional development	Teaching coordinators and school principals
43	What is the role of global health and sustainable development in Swedish medical education? A qualitative study of key stakeholders' perspectives	Velin et al.	2023	Sweden	Tertiary	Medical educators
44	A global educational experience between geography and nursing: Interdisciplinarity and sustainability	Constantinou & Morrison‐Beedy,	2021	Cyprus	Tertiary	Geography, Nursing
45	Exploring innovative strategies in problem based learning to contribute to sustainable development: A case study	Llach & Bastida	2023	Spain	Tertiary	Human biology students
46	Evaluation of the spirit integrated suicide prevention programme: Study protocol for a cluster‐randomised controlled trial in rural Gujarat, India.	Pathare et al.	2020	India	Vocational	Community health workers
47	Impact of COVID‐19 on academic and psychological aspects in students of medicine: A cross‐sectional study	Singla et al.	2023	India		

### Evaluation methodology

In terms of methodology, of the 11 papers containing a formal evaluation, four (36.36%) used exclusively qualitative methods to evaluate the effectiveness of the teaching method or intervention, five used quantitative methods (45.45%), and two used mixed methods (18.18%). Of those papers, four were non‐randomized studies.

### Population and location

Eleven papers (23.40%) focused specifically on training nurses and midwives, including trainees; one on medical students; three on psychologists; two on community health workers; one on emergency first responders; one on speech language therapists; and five on social workers. Eleven studies focused explicitly on training educators, including university academics and school teachers. Six studies (12.77%) referred to the health‐care workforce, broadly defined, and four studies did not specify a population.

With respect to location, the majority of studies were in the United States or did not specify a location (*n* = 8 for both), and a further seven (14.89%) described various locations, two described multiple countries in Africa, and one concerned low–middle income countries broadly. Most countries in the sample only had one study in that location, with exceptions being Australia (*n* = 4), Spain (*n* = 6), Sweden, Thailand, Norway, and India (all *n* = 2), and Uruguay (*n* = 1).

### SDG focus

**FIGURE 2:**
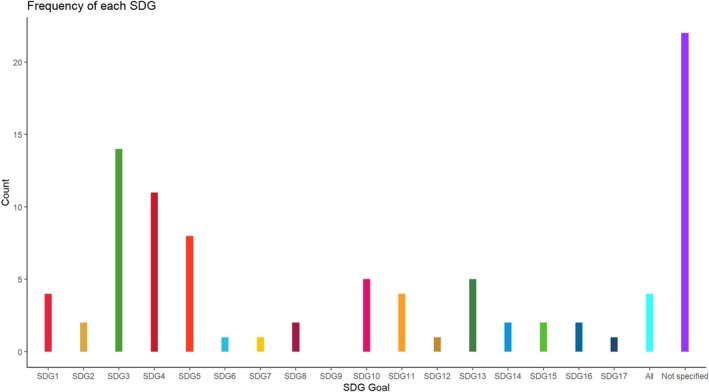
SDG focus of papers in the review. *Note.* Some papers addressed more than one SDG; hence, the total exceeds 47. SDG, sustainable development goals.

Figure 2 shows the specific SDGs addressed in the papers, based on explicit mention of individual SDGs within the text. Twenty‐three papers referred to the SDGs in general terms without further specification. In the remaining papers, SDG 3 (good health and well‐being) and SDG 4 (quality education) were the most commonly cited. Only one SDG (9; industry, innovation, and infrastructure) remained unaddressed across all papers, while four papers explicitly addressed all SDGs.

### Quality assessment

For the 12 papers that included an empirical evaluation, we conducted a quality assessment using the Mixed‐Methods Appraisal Tool. This can be accessed in Supporting Information File 1 on the Open Science Framework: https://osf.io/7xvqe/overview.

### Pedagogies and practice

Our QCA of the final papers was guided by our research question *“What is known from the literature about the pedagogies and practice of integrating the SDGs into education for mental health allied disciplines?”* and identified three broad approaches to pedagogy and practice in the reviewed papers: (1) *critical‐ and active‐learning* approaches, (2) *collaborative learning* approaches, and (3) *experiential and service‐learning* approaches. This QCA was conducted on the “mention of pedagogical strategies and practices” aspect of the data extraction table.

#### Critical and active learning

“Active learning,” an educational approach in which students engage with the material, participate in the learning process and take responsibility for their understanding of subject content, was mentioned explicitly in five of the reviewed papers (e.g., Fields et al., 2023; Sánchez De Miguel et al., 2022). Active learning involves activities like critical thinking, discussions, problem‐solving, and reflection, and is designed to contrast with passively receiving information from instructors. As a pedagogical principle, active learning emphasizes interaction and often incorporates group work, discussion of case studies, and inquiry‐based tasks. Many papers in the review highlighted pedagogical strategies, such as teamwork, group assessment, and collaboration, where students are encouraged to engage with course content through active and hands‐on interaction and practical applications to gain deeper understanding of the scope and purpose of the SDGs (e.g., Dieck‐Assad et al., 2021; McLean et al., 2022).

There were specific examples in the reviewed papers of practice to embed the SDGs. Flipped classrooms, where traditional teaching is inverted to focus more on discussion and problem‐solving during class time, were frequently mentioned (*n* = 3) alongside experiential and case‐based learning (*n* = 5; e.g., Bälter et al., 2022; Fields et al., 2023). Similarly, Burhin et al. (2023) found that the most effective teaching strategies involved a combination of theoretical sessions and hands‐on practical experiences, which helped students link theory with practice.

Additionally, some papers discuss innovative pedagogical practices, such as mind mapping (Kaloga & Reno, 2023) and conflict‐resolution strategies (Picado‐Valverde et al., 2022). Similarly, “problem‐based learning” was discussed explicitly as a principle that underpinned the practice of case studies (e.g., Llach & Bastida, 2023).

These methods aim to bridge the gap between theory and practice, fostering critical thinking and problem‐solving skills by immersing students in real‐world scenarios. Similarly, several papers explicitly prioritized active learning methodologies to engage students directly with content and encourage critical thinking. For instance, one paper discussed how the integration of planetary health into the medical curriculum emphasizes sustainability, empowering students to critically and actively analyze health‐care systems in the context of SDGs (McLean et al., 2022). In McLean et al. (2022), students examined the mental health implications of environmental stressors, using systems thinking to explore the intersections between mental well‐being, policy, and ecological change.

Similarly, a paper described a reimagined nursing capstone course that incorporates the SDG framework to foster transformative educational experiences for students, using active learning (Fields et al., 2023). Another example is the use of case studies in geriatric care to encourage reflection and the development of non‐prejudiced, stereotype‐free attitudes toward elderly patients (Sánchez De Miguel et al., 2022). Similarly, Suvarnabhumi et al. (2022) described an online module for family physicians that introduces sustainable development concepts to enhance their critical understanding of the SDGs. These initiatives share a common goal of cultivating critical, reflective learners through active engagement.

#### Collaborative learning

Collaborative practices and pedagogies also featured prominently (*n* = 8), with teamwork and group activities often highlighted as central to promoting peer learning and diversity of thought (Addo et al., 2023; Trubavina et al., 2021). We define collaborative learning as the process of students working together in groups or teams to solve problems, complete tasks, or create projects, often in interdisciplinary or cross‐cultural contexts. This approach highlights shared goals, mutual accountability, and the development of interpersonal skills.

Educational programs that promote collaborative learning also used practices, such as interdisciplinary engagement, partnership, and global collaboration, to achieve these goals. Other initiatives, such as global learning programs (GLPs), create opportunities for intercultural collaboration and rely upon smaller group interactions and interdisciplinary discussions (Gopal et al., 2023). Gopal et al. (2023) detailed a cross‐cultural training program in psychology that emphasized collaborative learning between institutions in different regions, with a focus on building cultural responsiveness and communication skills essential for mental health practitioners. These approaches align with global citizenship frameworks by promoting competence, responsibility, and civic engagement in addressing societal challenges (Sonn et al., 2021).

For example, papers emphasized collaborative learning with a focus on cross‐disciplinary and cross‐cultural partnerships to achieve the SDGs. Some interdisciplinary projects pair students from different cohorts to work on SDG‐related projects, such as analyzing health and well‐being in Cyprus (Constantinou & Morrison‐Beedy, 2021). Similarly, King et al. (2021) describe a nursing program that links United States and Norwegian students in community health nursing courses to develop global clinical experiences. A shared learning experience in Nepal also brought together students from multiple health disciplines to collaborate on workshops and community‐based initiatives (Lees et al., 2016).

Additionally, cross‐national programs in health service psychology connected Indian and U.S. institutions to revise curricula and promote professional development for sustainable development (Gopal et al., 2023). These examples highlight the importance of partnerships and teamwork in broadening educational horizons and enhancing interdisciplinary understanding; for example, Gopal et al. (2023) describe the benefit of international partnership and interactions with cross‐cultural teams to foster intercultural awareness and “meaningful and productive experiences.”

Collaborative learning, in some instances, was promoted through service‐learning and global learning partnerships, which integrate technology and web‐based platforms (Ezezika & Jarrah, 2022). These approaches reflect a growing trend toward incorporating digital tools and global perspectives into the learning process, enabling remote collaboration and broadening educational experiences. For example, Bälter et al. (2022) evaluated the OneLearns program, finding that the use of a digital platform to integrate theoretical and practical learning experiences was effective in supporting student engagement and development. This also included collaboration between staff and students. For example, Fields et al. (2023) recommended co‐creating knowledge with students and fostering dialogue, especially in interdisciplinary contexts, to enhance the learning experience. Srisaeng and Upvall (2020) reported on a follow‐up evaluation conducted 4 months after implementing a new teaching method, noting significant improvements in student confidence and knowledge retention. Srisaeng and Upvall (2020) described a midwifery education model that included hands‐on mental health training focused on supporting women and families during the perinatal period. These examples highlight how pedagogical approaches were not only theoretical but also actively shaped students' competencies in mental health‐care delivery, cultural awareness, and interdisciplinary practice.

#### Experiential and service learning

Our review also found papers that promote *experiential* learning between disciplines to realize the SDGs (*n* = 5; e.g., geography and nursing; Constantinou & Morrison‐Beedy, 2021). Experiential learning is, broadly, a hands‐on approach where students learn by doing and reflecting on their experiences. Rooted in real‐world contexts, it integrates practice and theory that encourages students to apply their knowledge in authentic scenarios to develop practical skills and deepen understanding. Service learning was also cited as a pedagogical approach to contribute to the SDGs (Mangas et al., 2021; Albareda‐Tiana et al., 2018). Service‐learning methodologies enabled students to critically reflect on societal issues, integrating communication, critical thinking, and self‐assurance skills into their learning processes (Castro et al., 2020). Community‐based learning opportunities were also highlighted (Castro et al., 2020; Upvall & Luzincourt, 2019). Experiential learning initiatives further grounded students in applied mental health settings. For instance, Lees et al. (2016) reported on a program where students from multiple health disciplines worked together on community‐based mental health education and outreach in Nepal.

Experiential learning programs prioritize hands‐on, real‐world applications of knowledge, often in settings outside of the university context. Dieck‐Assad et al. (2021), for example, describe a project in which students are trained as emergency first responders, providing direct experience with medical emergencies and health‐care awareness for SDG promotion (Dieck‐Assad et al., 2021). The Female Community Health Volunteer program in Nepal also promoted experiential learning by engaging students in practical health‐care delivery and behavior change communication (Schwarz et al., 2020). Similarly, papers described field‐based education initiatives that are designed to enable students to gain practical skills in real‐world, service‐learning contexts (Shatberashvili & Sadzaglishvili, 2022). WHO case studies also took this approach; for example, a description and evaluation of a nursing program in Australia that addresses workforce needs by combining postgraduate education with practical mental health service experiences to work toward the SDGs (World Health Organization, 2017). These programs show the value of practical engagement in preparing students for professional challenges.

In the papers we reviewed, experiential learning initiatives were described to provide hands‐on, real‐world applications of knowledge. For example, one midwifery education curriculum integrated practical teaching and clinical skills, enabling midwife educators to train others effectively in pursuit of service learning for the SDGs (Srisaeng & Upvall, 2020). Similarly, service‐learning projects that focused on evaluating the social impact of interventions while fostering personal responsibility norms were also included, again in the context of achieving sustainable development (Mangas et al., 2021). These programs aimed, broadly, to bridge the gap between theory and practice, preparing students for professional and societal contributions.

In many cases, the desire for experiential learning opportunities was grounded in specific critical pedagogies. We define critical pedagogies as educational approaches that focus on empowering students to critically analyze and challenge societal structures, power dynamics, and injustices (as per Freire, 1970). For example, Fields et al. (2023) explicitly refer to Freire and critical pedagogy, and outline how nursing students can be engaged with the SDGs through an undergraduate nursing course that prioritizes enlightenment (through awareness raising and dialog) to work toward empowerment (e.g., problem‐posing, praxis, meaning projects). In other words, this approach demonstrates how nursing education can integrate the SDGs by fostering awareness and critical engagement, ultimately empowering students to drive meaningful change. This approach is centered around raising critical consciousness (i.e., the ability to recognize and challenge social injustices through reflection and action). This encourages students to consider how economic, cultural, and systemic factors shape access to care and patient outcomes. Further, education through critical pedagogy encourages students to critique societal power structures and work toward SDGs, using dialogic and problem‐posing strategies (Fields et al., 2021, 2023). Similarly, Fox et al. (2018) highlighted the importance of promoting security and setting clear expectations within learning environments to ensure that students feel supported and engaged throughout the learning process.

### Challenges

To consider the effectiveness of pedagogies and practices, we also conducted QCA on the “proposed/experienced challenges and opportunities” part of the data extraction table.

#### Logistical challenges

The challenges faced in the reviewed studies highlight several recurring issues. Integrating large frameworks, such as the Mental Health Gap Action Program, proved difficult, particularly when trying to adopt extensive guidelines into existing training programs. Logistical constraints, including a lack of support, resources, and internet connectivity, hindered the implementation and delivery of some educational initiatives (e.g., Burhin et al., 2023; WHO, 2021). Clarification of roles within the health‐care workforce posed another challenge, as did the intensive and brief nature of some training programs, which limited the depth of learning. The absence of supervision and ongoing training was also a significant issue, particularly in health‐care education (e.g., Schwarz et al., 2020). Finally, issues, such as management of the emotional aspect of engaging with SDGs, from both a student and educator point of view, were also noted as challenges in this area (e.g., Llach & Bastida, 2023).

#### Cultural challenges

Some papers reflected upon how the SDGs are perceived to be based on Western knowledge and priorities, which can be a challenge for integration in contexts outside of the West. For example, the participants noted that a social work curriculum to develop SDG awareness should integrate indigenous or critical ways of knowing into the program to overcome this (Nnama‐Okechukwu et al., 2023). Additionally, many studies emphasized that understanding the local context was crucial for successful program implementation, yet this was often overlooked (e.g., Staley et al., 2023). For example, Mangas et al. (2021) noted how there was an inherent tension between personal responsibility and collective efforts for the SDGs. With this in mind, engaging stakeholders also presented difficulties, as their perspectives on opportunities and challenges were sometimes diverse and conflicting (e.g., Kunaviktikul & Turale, 2020).

#### Institutional challenges

Further, ensuring the sustainability of programs beyond the initial implementation phase was discussed as a challenge for some programs, particularly in terms of continued evaluation and support. Research‐related challenges included students being ill‐prepared for the research components of their courses and the changing role of universities (e.g., Neary & Osborne, 2018), particularly in a post‐COVID context (Sonn et al., 2021). There were also challenges related to gathering consensus about the value of different practitioner roles. For example, Velin et al. (2023) describe contentions surrounding the “modernization” of the medical profession to include awareness of the SDGs.

## DISCUSSION

This scoping review examined pedagogical approaches and practices for integrating the SDGs into interdisciplinary mental health education across various disciplines and in different cultural and geographical contexts. Three primary approaches were noted by our qualitative content analysis: *critical and active learning, collaborative learning*, and *experiential and service learnin*g. These approaches all aim to foster students' critical thinking, interdisciplinary collaboration, and hands‐on experiences, aligning education with the objectives of the breadth of the SDGs. Notably, these pedagogies are well established within global citizenship education literature and thus provide an important bridge to mental health education by offering practical approaches for embedding global responsibility, sustainability, and social justice within professional training. Challenges to meaningful implementation included logistical, cultural, and institutional barriers, such as resource limitations, Western‐centric frameworks, and sustainability of initiatives. Importantly, the review also shows how the existing body of literature is predominantly developmental in nature, with a relative paucity of outcome‐focused evidence, and this should be considered when interpreting the findings.

The results of this scoping review demonstrate the potential value of adopting a cross disciplinary lens to share pedagogical insights and practices in disciplines contributing to mental health education (see also Podgórska & Zdonek, 2024). By integrating the SDGs into interdisciplinary learning, educators can draw from diverse fields, fostering innovative approaches that enhance both teaching and learning (as per Herzig Van Wees et al., 2019). The shared emphasis on critical thinking, collaboration, and experiential learning across disciplines highlights the common ground in addressing mental health challenges while accommodating unique disciplinary perspectives. This interconnected approach not only strengthens the capacity of students to work collaboratively in solving complex mental health issues but also encourages a richer exchange of ideas and methods, ultimately promoting more holistic and inclusive educational practices. These findings emphasise the potential for cross disciplinary learning to break silos, build synergies, and equip future professionals with the skills needed to address the multifaceted nature of mental health within the broader framework of sustainable development (e.g., Guo, 2014; Pallant et al., 2020). Interdisciplinary learning may, more broadly, be necessary to advance the SDGs in other contexts too (Andreoni & Richard, 2024; Podgórska & Zdonek, 2024).

While these pedagogical approaches emerged across disciplines, their implementation in mental health education reveals important patterns and tensions. For example, critical and active learning strategies were often used to engage students in questioning the social determinants of mental health, aligning closely with SDGs such as SDG3 (Good Health and Well‐Being) and SDG10 (Reduced Inequalities). However, such approaches were often delivered through one‐off modules or short‐term interventions, limiting their sustained impact. Collaborative learning was frequently framed through interprofessional education, supporting SDG17 (Partnerships for the Goals), yet the reviewed studies showed uneven success in fostering true interdisciplinary exchange, often constrained by institutional silos. Experiential and service learning offered some of the most direct engagements with SDG‐linked outcomes, particularly through placements or community‐based projects, but these too were limited by resource disparities and challenges in sustaining long‐term partnerships. Thus, while the pedagogies identified have strong conceptual alignment with SDG integration, their practical effectiveness is often undermined by logistical and structural barriers, highlighting a gap between pedagogical intent and systemic implementation in mental health education.

Furthermore, while the identification of critical and active learning, collaborative learning, and experiential/service learning provides a high‐level typology, our analysis also revealed how these pedagogies are specifically applied and adapted within mental health education. For instance, active learning in nursing education was often underpinned by critical pedagogies focused on structural determinants of mental health (e.g., Fields et al., 2023), with students engaging in reflective exercises that linked SDG3 (Good Health and Well‐Being) to lived experiences of health inequity. Similarly, collaborative learning was not merely interpersonal, but frequently interdisciplinary, reflecting the realities of mental health workforce training. Programs like those described by Gopal et al. (2023) used interprofessional dialogue and cross‐cultural case discussions to develop competencies in culturally safe care and global mental health literacy, directly aligning with SDGs 3, 4, and 17. Experiential and service learning initiatives, such as those embedded in midwifery and psychology curricula (e.g., Lees et al., 2016; Srisaeng & Upvall, 2020), engaged students in real‐world placements and community engagement projects focused on trauma‐informed practice, disaster response, and social justice. These examples illustrate that while pedagogical categories may appear broad, their implementation is often tightly connected to the distinctive needs of mental health education and the competencies required to advance SDG‐relevant outcomes.

However, our review highlights the need for more robust frameworks and strategies to overcome these barriers and streamline SDG integration in education of the future mental health workforce. By including diverse studies and educational practices, the review provides more nuanced and interdisciplinary insights into how SDGs can be embedded in mental health education. This was a comprehensive review, spanning multiple disciplines and global contexts, and extending previous reviews that have existed within disciplinary silos and, as such, have not looked across disciplines and subjects at opportunities for shared learning. However, only 47 papers were found within the review context, and among those papers, only 12 contained an evaluation element. This suggests, broadly, that more research is needed into pedagogies and practices used to embed the SDGs into mental health workforce education. In addition to quantity, clearer testing and reporting of program outcomes would help fellow educators to understand how SDGs have been implemented in similar contexts and how successful that implementation was. Many of the reviewed papers discussed integrating SDGs but did not specify either the SDG (45.8%) or the pedagogical strategy used. Even if evaluation is not possible for logistical reasons, being more explicit within reporting of case studies and educational programs would help educators to better understand how they can integrate such programs themselves.

Some important notes from our QCA of the challenges educators experienced are the difficulty of implementing programs, such as the WHO Mental Health Gap Action Program and the Western nature of the SDGs. Both are intended to have global relevance and to be adaptable across cultures. However, that the studies reviewed found these challenging suggests a need for further consideration on how SDGs and the existing frameworks intended to achieve them relate to non‐Western contexts. This is particularly important when educating the mental health workforce, given cross‐cultural differences in the expression and treatment of mental health.

### Implications

The findings of this scoping review have important implications for mental health education and workforce development. Notably, a substantial proportion of the included studies were able to explicitly identify pedagogical principles underpinning the integration of the SDGs, suggesting that this area of education is already grounded in well‐established educational theory. This theoretical coherence is a key strength of the existing literature, indicating that educators are not approaching SDG integration in an ad hoc manner but are instead drawing on recognized pedagogical frameworks, including those associated with global citizenship education and education for sustainable development (Horey et al., 2018; Kioupi & Voulvoulis 2019; Pownall et al., 2024), which are distinct but allied frameworks. The consistency with which these pedagogical principles were articulated across diverse disciplines and contexts suggests that the conceptual foundations for SDG‐integrated mental health education are largely in place, and this review is the first to identify this state of the evidence.

At the same time, the gaps identified in the literature appear to be predominantly *practical* rather than *pedagogical*. Challenges related to resources, institutional support, curriculum constraints, and the sustainability of initiatives were commonly reported, despite broad agreement on appropriate teaching approaches. This implies that the key task for advancing SDG integration in mental health education in the future is not the development of new pedagogical *theory*, but rather the translation of existing theory into scalable, context‐sensitive, and institutionally supported *practices*. Future efforts may therefore benefit from shifting focus toward implementation strategies, faculty development, and structural enablers that allow theoretically sound pedagogies to be embedded within routine mental health training. In this sense, the literature suggests that the question is no longer what pedagogies are needed, but how these pedagogies can be made feasible and sustainable in practice.

### Limitations

The review has some limitations, however. First, the exclusion of non‐English studies may have omitted valuable insights from non‐Western contexts, potentially skewing the findings toward Western‐centric perspectives. Indeed, many of the studies included in our review are from high‐income Western countries or papers that do not clearly specify their cultural context. This means that we are unable to precisely identify whether the pedagogies involved in training the mental health workforce may differ across cultural, institutional, and linguistic settings. Future work should thus consider the core and adaptable components of mental health pedagogies, while also identifying the pedagogies that are locally or culturally specific.

Additionally, the reliance on published and peer‐reviewed sources may have overlooked gray literature, limiting the review's ability to capture grassroots or innovative educational practices not widely disseminated. For example, we did not examine Open Educational Resources, policy papers, or more informal summaries of attempts to implement the SDGs in disciplinary contexts. Further, another limitation is how only a small number of the reviewed papers included an evaluation of the practice or pedagogy. This means that, while we can explore how educators intend to integrate the SDGs across disciplines, little is known about how effective such efforts are. This, while this review demonstrates emerging pedagogical directions and conceptual innovations, empirical evidence regarding long‐term effectiveness and educational outcomes remains limited.

Future work may wish to examine non‐English contributions, with robust evaluations to explore this further. However, this lack of evaluation also points to a need for more studies within this field that test the effectiveness of mental health workforce education in implementing SDGs. Finally, we took an intentionally broad approach to defining the population of the mental health workforce (and included, for example, religious leaders and first responders, as well as students). Although a broad interdisciplinary definition aligns with holistic approaches to mental health promotion across cultures, clearer conceptual differentiation between specialist mental health professions and broader well‐being–oriented educational roles may have strengthened analytical and conceptual precision. Future work may also focus in on a tighter definition of the front‐line mental health workforce.

## CONCLUSION

This review explored the pedagogies and practice of educators in advancing the SDGs within mental health disciplines. By highlighting effective pedagogies and practices, this paper aims to provide a roadmap for educators and institutions to embed sustainability into curricula. Addressing the identified challenges, particularly resource disparities and cultural relevance, will be essential for maximizing the impact of SDG‐oriented education. Future research should focus on evaluating long‐term outcomes and expanding the scope to include non‐English and underrepresented contexts. This will ensure that SDG integration in mental health education is both comprehensive and globally inclusive.

## CONFLICT OF INTEREST STATEMENT

The authors declare no conflicts of interest.

## ETHICS STATEMENT

Ethical approval was not required for this study as it is a systematic review that synthesizes existing literature and does not involve the collection or presentation of new primary data.

## Data Availability

Data Data extraction can be openly accessed in the project Open Science Framework page https://osf.io/7xvqe/overview.

## References

[aphw70192-bib-0001] Addo, R. , Koers, G. , & Timpson, W. M. (2023). Teaching sustainable development goals and social development: A case study teaching method. Social Work Education, 41, 1478–1488. 10.1080/02615479.2022.2112168

[aphw70192-bib-0002] Adebayo, Y. O. , Adesiyan, R. E. , Amadi, C. S. , Ipede, O. , Karakitie, L. O. , & Adebayo, K. T. (2024). Cross‐cultural perspectives on mental health: Understanding variations and promoting cultural competence. World Journal of Advanced Research and Reviews, 23(01), 432–439.

[aphw70192-bib-0003] Aktas, F. , Pitts, K. , Richards, J. C. , & Silova, I. (2017). Institutionalizing global citizenship: A critical analysis of higher education programs and curricula. Journal of Studies in International Education, 21(1), 65–80.

[aphw70192-bib-0004] Albareda‐Tiana, S. , Vidal‐Ramentol, S. , & Fernández‐Morilla, M. (2018). Implementing the sustainable development goals at university level. International Journal of Sustainability in Higher Education, 19, 473–497. 10.1108/ijshe-05-2017-0069

[aphw70192-bib-0005] Andreoni, V. , & Richard, A. (2024). Exploring the interconnected nature of the sustainable development goals: the 2030 SDGs Game as a pedagogical tool for interdisciplinary education. International Journal of Sustainability in Higher Education, 25(1), 21–42. 10.1108/IJSHE-11-2022-0378

[aphw70192-bib-0006] Assarroudi, A. , Heshmati Nabavi, F. , Armat, M. R. , Ebadi, A. , & Vaismoradi, M. (2018). Directed qualitative content analysis: The description and elaboration of its underpinning methods and data analysis process. Journal of Research in Nursing, 23(1), 42–55. 10.1177/1744987117741667 34394406 PMC7932246

[aphw70192-bib-0007] Australian Institute of Health and Welfare . (2023). Workforce—Mental health https://www.aihw.gov.au/mental-health/topic-areas/workforce#Specialist_workers

[aphw70192-bib-0008] Badawy, W. , & Shaban, M. (2024). The role of nursing education in advancing sustainable development goals: A rapid review of current pedagogical strategies. Teaching and Learning in Nursing Advance Online Publication. 10.1016/j.teln.2024.10.014

[aphw70192-bib-0009] Bälter, K. , Abraham, F. J. , Mutimukwe, C. , Mugisha, R. , Osowski, C. P. , & Bälter, O. (2022). A web‐based program about sustainable development goals focusing on digital learning, digital health literacy, and nutrition for professional development in Ethiopia and Rwanda: Development of a pedagogical method. JMIR Formative Research, 7, e36585. 10.2196/36585 PMC9764148 36469416

[aphw70192-bib-0010] Beagon, U. , Kövesi, K. , Tabas, B. , Nørgaard, B. , Lehtinen, R. , Bowe, B. , … Spliid, C. M. (2023). Preparing engineering students for the challenges of the SDGs: What competences are required? European Journal of Engineering Education, 48(1), 1–23.

[aphw70192-bib-0011] British Psychological Society . (2024). Standards for the accreditation of undergraduate, conversion and integrated master's programmes in psychology. The British Psychological Society.

[aphw70192-bib-0012] Burhin, M. , Isom, V. , Ogaoga, D. , Devine, S. , Duke, T. , Bugoro, H. , Tamou, M. , Mark, C. , & Panda, N. (2023). Child health nurses in Solomon Islands, piloting the ‘Bachelor of Nursing: Child Health’. International Nursing Review, 71, 44–53. 10.1111/inr.12836 37029778

[aphw70192-bib-0013] Burns, H. L. , Kelley, S. S. , & Spalding, H. E. (2019). Teaching sustainability: Recommendations for best pedagogical practices. Journal of Sustainability Education.

[aphw70192-bib-0014] Castro, P. M. , Ares‐Pernas, A. , & Dapena, A. (2020). Service‐learning projects in university degrees based on sustainable development goals: Proposals and results. Sustainability, 12, 7940. 10.3390/su12197940

[aphw70192-bib-0015] Constantinou, S. T. , & Morrison‐Beedy, D. (2021). A global educational experience between geography and nursing: Interdisciplinarity and sustainability. The Geography Teacher, 18, 142–145. 10.1080/19338341.2021.1931923

[aphw70192-bib-0016] Cranney, J. , Morris, S. , Norris, K. , & Connolly, C. E. (2022). Charting the psychological literacy landscape: Systematic review highlighting psychology education. Frontiers in Education, 7. 10.3389/feduc.2022.913814

[aphw70192-bib-0017] Dieck‐Assad, G. , Pena, O. I. G. , & Rodriguez‐Delgado, J. M. (2021). Evaluation of emergency first response's competency in undergraduate college students: Enhancing sustainable medical education in the community for work occupational safety. International Journal of Environmental Research and Public Health, 18, 7814. 10.3390/ijerph18157814 34360107 PMC8345564

[aphw70192-bib-0018] Edwards, D. B. Jr. , Sustarsic, M. , Chiba, M. , McCormick, M. , Goo, M. , & Perriton, S. (2020). Achieving and monitoring education for sustainable development and global citizenship: A systematic review of the literature. Sustainability, 12(4), 1383. 10.3390/su12041383

[aphw70192-bib-0019] Ezezika, O. , & Jarrah, M. (2022). A case study on the impact of a web‐based animated storyline module for global health pedagogy: Student perspectives. Journal of Education, 202(1), 26–33. 10.1177/0022057420943188

[aphw70192-bib-0021] Fields, L. , Moroney, T. , Perkiss, S. , & Dean, B. A. (2023). Enlightening and empowering students to take action: Embedding sustainability into nursing curriculum. Journal of Professional Nursing, 49, 57–63. 10.1016/j.profnurs.2023.09.001 38042563

[aphw70192-bib-0022] Fields, L. , Perkiss, S. , Dean, B. A. , & Moroney, T. (2021). Nursing and the sustainable development goals: A scoping review. Journal of Nursing Scholarship, 53(5), 568–577. 10.1111/jnu.12675 34056841

[aphw70192-bib-0023] Fox, J. , Gupta, R. , & Mitchell, G. (2018). Considering developmental concepts from attachment theory to inform graduate student training in global trauma and disaster psychology. International Perspectives in Psychology, 7, 189–201. 10.1037/ipp0000092

[aphw70192-bib-0024] Freire, P. (1970). Pedagogy of the oppressed. Continuum.

[aphw70192-bib-0025] Gopal, B. , Burlew, A. K. , D'Souza, G. , George, T. S. , Varghese, K. J. , & Raval, V. V. (2023). Facilitating faculty development for training in multicultural competence in health service psychology graduate programs through an international collaboration. International Journal of Psychology and Education. 10.1027/2157-3891/a000089

[aphw70192-bib-0026] Guo, L. (2014). Preparing teachers to educate for 21st century global citizenship: Envisioning and enacting. Journal of Global Citizenship & Equity Education, 4(1), 1–23.

[aphw70192-bib-0027] Herzig Van Wees, S. L. , Målqvist, M. , & Irwin, R. (2019). Achieving the SDGs through interdisciplinary research in global health. Scandinavian Journal of Public Health, 47(8), 793–795. 10.1177/1403494818812637 30486761

[aphw70192-bib-0028] Horey, D. , Fortune, T. , Nicolacopoulos, T. , Kashima, E. , & Mathisen, B. (2018). Global citizenship and higher education: A scoping review of the empirical evidence. Journal of Studies in International Education, 22(5), 472–492. 10.1177/1028315318786443

[aphw70192-bib-0029] Hsieh, H. F. , & Shannon, S. E. (2005). Three approaches to qualitative content analysis. Qualitative Health Research, 15(9), 1277–1288. 10.1177/1049732305276687 16204405

[aphw70192-bib-0030] Kaloga, M. , & Reno, R. (2023). SDG's and systems science: Teaching beyond the micro‐macro divide in social work education. Social Work Education, 41, 1489–1506. 10.1080/02615479.2022.2109622

[aphw70192-bib-0031] King, T. S. , Bochenek, J. , Jenssen, U. , Bowles, W. , & Morrison‐Beedy, D. (2021). Virtual study‐abroad through web conferencing: Sharing knowledge and building cultural appreciation in nursing education and practice. Journal of Transcultural Nursing, 32, 790–798. 10.1177/10436596211009583 33855909

[aphw70192-bib-0063] Kioupi, V. , & Voulvoulis, N. (2019). Education for sustainable development: A systemic framework for connecting the SDGs to educational outcomes. Sustainability, 11(21), 6104.

[aphw70192-bib-0032] Kunaviktikul, W. , & Turale, S. (2020). Internationalizing nursing curricula in a rapidly globalizing world. Nursing Education Review. 10.1016/j.nepr.2020.102704 31991380

[aphw70192-bib-0033] Lees, J. , Webb, G. , Coulston, F. , Smart, A. , & Remedios, L. (2016). Health professional workforce education in the Asia Pacific. Journal of Public Health Research, 5(1), jphr‐2016. 10.4081/jphr.2016.658 PMC4856868 27190976

[aphw70192-bib-0034] Leite, S. (2022). Using the SDGs for global citizenship education: Definitions, challenges, and opportunities. Globalisation, Societies and Education, 20(3), 401–413. 10.1080/14767724.2021.1882957

[aphw70192-bib-0035] Llach, M. C. , & Bastida, M. L. (2023). Exploring innovative strategies in problem‐based learning to contribute to sustainable development: A case study. Educational Innovations Quarterly.

[aphw70192-bib-0036] Mangas, S. , Marbán, J. M. , Unanue Cuesta, M. C. , Manso Argüelles, M. Á. , & Romay Martínez, J. (2021). The role personal responsibility norms play in sustainable development for university students: The impact of service‐learning projects. Sustainability, 13(13), 7330. 10.3390/su13137330

[aphw70192-bib-0037] McLean, M. , Phelps, C. , Smith, J. , Maheshwari, N. , Veer, V. , Bushell, D. , … Moro, C. (2022). An authentic learner‐centered planetary health assignment: A five‐year evaluation of student choices to address sustainable development goal 13 (climate action). Frontiers in Public Health, 10, 1049932. 10.3389/fpubh.2022.1049932 36408043 PMC9671629

[aphw70192-bib-0038] Mills, C. (2018). From ‘invisible problem’ to global priority: The inclusion of mental health in the sustainable development goals. Development and Change, 49(3), 843–866. 10.1111/dech.12397

[aphw70192-bib-0039] Neary, J. , & Osborne, M. (2018). University engagement in achieving sustainable development goals: A synthesis of case studies from the SUEUAA study. Australian Journal of Adult Learning, 58(3), 336–364.

[aphw70192-bib-0040] Nnama‐Okechukwu, C. , McLaughlin, H. , Okoye, U. , Hendricks, E. , Imaan, L. , Malinga, T. , … Imo, N. (2023). Indigenous knowledge and social work education in Nigeria: Challenges and need for sustainable development. International Social Work, 66(6), 1857–1871. 10.1177/00208728221098511

[aphw70192-bib-0041] Pallant, E. , Choate, B. , & Haywood, B. (2020). How do you teach undergraduate university students to contribute to UN SDGs 2030? In Universities as living labs for sustainable development: supporting the implementation of the sustainable development goals (pp. 69–85).

[aphw70192-bib-0042] Patel, V. , Saxena, S. , Lund, C. , Thornicroft, G. , Baingana, F. , Bolton, P. , Chisholm, D. , Collins, P. Y. , Cooper, J. L. , Eaton, J. , Herrman, H. , Herzallah, M. M. , Huang, Y. , Jordans, M. J. D. , Kleinman, A. , Medina‐Mora, M. E. , Morgan, E. , Niaz, U. , Omigbodun, O. , … UnÜtzer, J. (2018). The Lancet commission on global mental health and sustainable development. The Lancet (British Edition), 392(10157), 1553–1598. 10.1016/S0140-6736(18)31612-X 30314863

[aphw70192-bib-0043] Picado‐Valverde, E. M. , Yurrebaso, A. , Guzmán‐Ordaz, R. , Nieto‐Librero, A. B. , & Gonzalez‐García, N. (2022). Approach developed according to sustainable development goals and challenges for future professionals in social intervention. Social Sciences, 11(2), 67. 10.3390/socsci11020067

[aphw70192-bib-0044] Podgórska, M. , & Zdonek, I. (2024). Interdisciplinary collaboration in higher education towards sustainable development. Sustainable Development, 32(3), 2085–2103. 10.1002/sd.2765

[aphw70192-bib-0045] Pownall, M. , Birtill, P. , & Harris, R. (2024). Student perceptions of global citizenship education in the university curriculum. Perspectives: Policy and Practice in Higher Education, 1–10.

[aphw70192-bib-0046] Sánchez De Miguel, M. , Orkaizagirre‐Gomara, A. , Izagirre‐Otaegi, A. , Ortiz de Elguea‐Díaz, F. J. , Badiola‐Etxaburu, I. , & Gómez‐Gastiasoro, A. (2022). Development, application and evaluation of an active learning methodology for health science students, oriented towards equity and cultural diversity in the treatment and care of geriatric patients. International Journal of Environmental Research and Public Health, 19(21), 14573. 10.3390/ijerph192114573 36361451 PMC9656949

[aphw70192-bib-0047] Schwarz, R. , Thapa, A. , Sharma, S. , & Kalaunee, S. P. (2020). At a crossroads: How can Nepal enhance its community health care system to achieve sustainable development goal 3 and universal health coverage? Journal of Global Health, 10(1), 010309. 10.7189/jogh.10.010309 32257137 PMC7100858

[aphw70192-bib-0048] Shatberashvili, N. , & Sadzaglishvili, S. (2022). SDG‐focused social work education in Georgia. Social Work Education, 41(7), 1507–1524. 10.1080/02615479.2022.2103111

[aphw70192-bib-0049] Sonn, I. K. , Du Plessis, M. , Jansen Van Vuuren, C. D. , Marais, J. , Wagener, E. , & Roman, N. V. (2021). Achievements and challenges for higher education during the COVID‐19 pandemic: A rapid review of media in Africa. International Journal of Environmental Research and Public Health, 18(24), 12888. 10.3390/ijerph182412888 34948499 PMC8700769

[aphw70192-bib-0050] Srisaeng, P. , & Upvall, M. J. (2020). Looking toward 2030: Strengthening midwifery education through regional partnerships. Journal of Advanced Nursing, 76(2), 715–724. 10.1111/jan.14015 30937943

[aphw70192-bib-0051] Staley, B. , Hickey, E. , Gibson, R. , Rochus, D. , & Nafukho, M. (2023). Obulala Na‐maani: Unity is strength: Speech‐language therapy and community engagement in three Kenyan communities. In handbook of speech‐language therapy in sub‐Saharan Africa: Integrating research and practice (pp. 197–211). Springer International Publishing. 10.1007/978-3-031-04504-2_10

[aphw70192-bib-0052] Suvarnabhumi, K. , Jorajit, S. , Dechpichai, W. , & Ativitthayaporn, J. (2022). Expert consensus on core topics of sustainable development online learning module for family physicians: A Delphi study. BMJ Open, 12(12), e061407. 10.1136/bmjopen-2022-061407 PMC9806043 36581405

[aphw70192-bib-0053] Trubavina, I. , Medvid, M. , Cwer, A. M. , Petryshyn, L. , & Meshko, H. (2021). Substantiation of the advanced training program “Social work with military personnel and military‐social work in the context of sustainable development goals”, E3S Web of Conferences (Vol. 280, p. 04007). EDP Sciences. 10.1051/e3sconf/202128004007

[aphw70192-bib-0054] UNESCO . (2020). Higher education figures at a glance. UNESCO Institute for Statistics. https://uis.unesco.org/sites/default/files/documents/f_unesco1015_brochure_web_en.pdf

[aphw70192-bib-0055] United Nations . (2015). The 17 sustainable development goals. United Nations. https://sdgs.un.org/goals

[aphw70192-bib-0056] Upvall, M. J. , & Luzincourt, G. (2019). Global citizens, healthy communities: Integrating the sustainable development goals into the nursing curriculum. Nursing Outlook, 67(6), 649–657. 10.1016/j.outlook.2019.04.004 31439322

[aphw70192-bib-0057] Velin, L. , Svensson, P. , Alfvén, T. , & Agardh, A. (2023). What is the role of global health and sustainable development in Swedish medical education? A qualitative study of key stakeholders' perspectives. BMC Medical Education, 23(1), 511. 10.1186/s12909-023-04502-y 37460947 PMC10353143

[aphw70192-bib-0058] World Health Organization . (2017). Optimizing the contributions of the nursing and midwifery workforce to achieve universal health coverage and the sustainable development goals through education, research and practice

[aphw70192-bib-0060] World Health Organization . (2021). Project on scaling up midwifery education in four African countries: Final project: Evaluation report.

[aphw70192-bib-0061] World Health Organization . (2022). World health statistics 2022. https://www.who.int/news/item/20-05-2022-world-health-statistics-2022

